# A gap between acceptance and knowledge of herbal remedies by physicians: The need for educational intervention

**DOI:** 10.1186/1472-6882-5-20

**Published:** 2005-11-18

**Authors:** Yuri N Clement, Arlene F Williams, Kristi Khan, Tricia Bernard, Savrina Bhola, Maurice Fortuné, Oneil Medupe, Kerry Nagee, Compton E Seaforth

**Affiliations:** 1 Faculty of Medical Sciences, The University of the West Indies, Trinidad and Tobago

## Abstract

**Background:**

The unprecedented global increase in the use of herbal remedies is set to continue apace well into the foreseeable future. This raises important public health concerns, especially as it relates to safety issues including adverse effects and herb-drug interactions. Most Western-trained physicians are ignorant of the risks and benefits of this healthcare modality and assessment of acceptance and knowledge would identify appropriate intervention strategies to improve physician-patient communication in this area.

**Methods:**

A cross-sectional survey was done using an interviewer-administered pilot tested *de novo *questionnaire at six public hospitals in Trinidad between May–July 2004. The questionnaire utilized weighed questions to quantify acceptance (maximum score = 14 points) and knowledge (maximum score = 52 points). Acceptance and knowledge scores were analyzed using the ANOVA and Tukey's tests.

**Results:**

Of 192 physicians interviewed, most (60.4%) believed that herbal remedies were beneficial to health. Respondents had relatively high acceptance levels (mean = 5.69 ± 0.29 points or 40% of total possible score) and poor knowledge (mean = 7.77 ± 0.56 points or 15% of total possible score). Seventy-eight physicians (40.6%) admitted having used herbs in the past, and 60 of these (76.9%) were satisfied with the outcome. Although 52 physicians (27.1%) recommended the use of herbs to their patients only 29 (15.1%) were able to identify at least one known herb-drug interaction.

**Conclusion:**

The use of herbal remedies is relatively high in Trinidad, as throughout the world, and most patients self-medicate with or without the knowledge of their attending physician. Surprisingly, we demonstrated relatively high acceptance levels and use of herbs among physicians in Trinidad. This interesting scenario of high acceptance levels and poor knowledge creates a situation that demands urgent intervention. We recommend educational intervention to narrow the gap between acceptance and knowledge so that physicians would be adequately equipped to communicate with their patients on this modality. The integration of herbal medicine into the curriculum of medical schools, continuing education programs and the availability of reputable pharmacopoeias for referencing at public health institutions are useful instruments that can be used to close this gap and promote improved physician-patient communication.

## Background

More than 80% of the population in the developing world use traditional medicine, which includes herbal remedies, for the management of health [1]. In the developed world the dramatic revolution in healthcare was facilitated by the discovery of pharmacologically active chemical entities (supported by evidence-based safety and efficacy testing) and this has shifted the emphasis away from traditional herbal medicine. Consequently, many Western-trained physicians have little formal training and lack knowledge on the benefits and risks of herbal remedies [2,3].

There has been an unprecedented explosion in the popularity of herbal preparations during the last few decades, especially in developed countries [4]. This phenomenon has stimulated considerable public health concern among physicians who are sometimes uncertain about the safety of herbs, especially when used concomitantly with allopathic drugs [5,6]. Despite these concerns, the global prevalence of use of medicinal herbs continues to rise as patients self-medicate with or without informing their physicians [1]. In this setting, the attitudes and knowledge of physicians would impact on the doctor-patient relationship and affect the overall quality of healthcare delivery, particularly with respect to issues such as possible adverse herb effects and herb-drug interactions [7-12].

There is significant use of herbal remedies in the Caribbean [13,14] and recent studies in Trinidad show relatively high prevalence of use for symptomatic relief in asthma [15] and therapeutic management in diabetes mellitus [16]. Merritt-Charles et al [17] reported an 86% lifetime prevalence of use among outpatients at a surgical facility on the island. This high prevalence of use dictates that the most appropriate intervention strategies be implemented to facilitate improved healthcare delivery, especially as it relates to physicians' knowledge of herbal medicines.

Few studies report on the knowledge, attitudes and practices of physicians regarding complementary and alternative medicine, with little emphasis on herbal medicine [18,19]. This study was undertaken primarily to determine the level of acceptance and knowledge regarding herbal medicine by physicians at public hospitals in Trinidad.

## Methods

This descriptive study was cross-sectional in design and used a *de novo *pilot-tested questionnaire during the period May to July 2004. Weighted questions were used to quantitatively assess physicians' acceptance and knowledge of herbal remedies. A sample of public health sector physicians was recruited from the six public hospitals in Trinidad. The nature and purpose of the study were explained on an individual basis, and following the physicians' willingness to participate they signed their informed consent. Respondents were permitted to withdraw from the study at anytime after the interview had commenced. The study was approved by the Ethics Committee, Faculty of Medical Sciences at the University of the West Indies, Trinidad.

### Setting and sample

The sample of physicians was recruited from the six public hospitals, namely the Port-of-Spain General Hospital (POSGH), the San Fernando General Hospital (SFGH) and the Eric Williams Medical Sciences Complex (EWMSC). These are the three major hospitals and employ most physicians in the public healthcare sector on the island. The other hospitals were the specialist obstetrics and gynecology Mount Hope Women's Hospital (MHWH), the St. Ann's Psychiatric Hospital (SAPH) and the minor Sangre Grande Regional Hospital (SGRH).

Quota sampling was used to obtain the sample size; the total number of physicians required was proportionally distributed among the hospitals based on the population of physicians at each hospital; i.e. the larger hospitals represented a larger percentage of the sample size. Furthermore, at these larger general hospitals, a representative proportion of physicians from all specialized department was obtained, and this was based on the total number of physicians working at each department. At these departments physicians were interviewed by convenient sampling until the quota was achieved. At the smaller specialist hospitals convenient sampling was used, without stratification, until the quota was achieved. With this method of interviewee selection we expected a 100% response rate. For inclusion in the study physicians must have been employed at the hospital for at least three months.

### Interview instrument

The pilot-tested *de novo *questionnaire was interviewer-administered and determined demographic details including gender, nationality, country of study, duration of employment at the hospital, position at the hospital, years of practicing medicine and level of qualification.

Acceptance (or positive attitude) was assessed using questions that evaluated beliefs, feelings and actions regarding herbal medicines (Appendix 1). Questions were developed *de novo *to assess these cognitive, evaluative and behavioural aspects of acceptance with a maximum possible score of 14 points. We rationalized that the behavioural component would be the best indicator of acceptance, as it is sometimes possible for beliefs and feelings to be discordant with actions. We estimated that the cognitive and evaluative aspects contributed less weight to acceptance and these items were assigned fewer points (4 out of 14 points). Items on behaviour included personal use, past recommendation and prescription of medicinal herbs to patients, and these were allocated a larger proportion of the overall points (10 out of 14 points).

Knowledge was evaluated using open-ended weighed questions to identify five (5) Caribbean and five (5) non-Caribbean medicinal herbs, their uses, contraindications and important herb-drug interactions, together with other questions determined knowledge of herbal pharmacopoeias and clinical studies on herbal medicines with a maximum score of 52 (Appendix 2). Established pharmacopoeias [20-22] and reputable websites [23,24] were used to determine correct responses related to medicinal herbs (Caribbean and non-Caribbean), their indications and contraindications. A database for important herb-drug interactions was compiled from published reviews [8,11,12].

### Statistical analysis

We postulated that most physicians in Trinidad would reject the use of herbal medicines, as did 82% of oncologists who rejected complementary and alternative medicines in the study by Hyodo et al [6]. We used this published prevalence rate to calculate a sample size of 192 physicians, with a confidence level of 95% [25]. This represented about 20% of physicians in the public health sector in Trinidad. The scores for acceptance and knowledge of herbal remedies were expressed as mean ± standard error of mean. ANOVA test was used to determine statistical significance between acceptance scores and knowledge scores and gender, nationality, country of study, years practicing medicine, hospital and department at hospital. Results were considered statistically significant when p < 0.05. In instances where p < 0.05, Tukey's test was used to determine statistical differences within and between groups. The data was analyzed using the Statistical Program for Social Sciences (SPSS) for Windows computer program (Version 11.0, Chicago, IL).

## Results

### Demography

One hundred and ninety two (192) survey questionnaires were completed (100% response rate) and the demographic details of the sample are given in Table 1. Most respondents were from the major hospitals (82.3%) and were male (74.0%). Most physicians were either native to the Caribbean or Latin America (62.5%) or trained in the region (55.2%). About one-third of the physicians interviewed were either nationals of, or trained in India/Asia and Nigeria. Most physicians (75.0%) had less than ten years medical practice experience. Most respondents (70.2%) were employed in the pediatrics, surgery, general medicine, obstetrics and gynecology and psychiatry departments at the hospitals.

**Table 1:** Demography and factors affecting knowledge and acceptance of herbal medicines of public health sector physicians in Trinidad

**Demographic factor**	**N (%)**	**Mean knowledge scores (maximum = 52)**	**Mean acceptance scores (maximum = 14)**
**Gender**			
Male	142 (74)	7.55 ± 0.65	5.60 ± 0.34
Female	50 (26)	8.40 ± 1.14	4.83 ± 0.57

**Years practicing medicine**			
Less than 5	95 (49.5)	7.68 ± 0.82	6.08 ± 0.40
6 to 10	49 (25.5)	6.84 ± 1.03	4.68 ± 0.56
More than 10	48 (25.0)	8.91 ± 1.20	5.95 ± 0.63

**Nationality**			
Caribbean/Latin American	120 (62.5)	9.48 ± 0.78 ^*1^	5.49 ± 0.36
Indian/Asian	47 (24.5)	4.42 ± 0.71	6.36 ± 0.68
Nigerian	21 (10.9)	4.76 ± 1.13	5.14 ± 0.68
European/North American	4 (2.1)	11.88 ± 4.72	6.75 ± 3.03

**Country of study**			
Caribbean/Latin America	106 (55.2)	9.24 ± 0.82 ^*2^	5.56 ± 0.39
India/Asia	48 (25.0)	4.69 ± 0.77	6.27 ± 0.65
Nigeria	22 (11.5)	4.77 ± 1.08	4.98 ± 0.67
Europe/North America	6 (3.1)	11.25 ± 3.91	8.58 ± 1.73
More than 1 country	10 (5.2)	11.50 ± 2.84	4.20 ± 1.19

**Hospital**			
SFGH	56 (29.2)	6.52 ± 0.91	4.67 ± 0.52
POSGH	58 (30.2)	7.85 ± 1.11	5.41 ± 0.49
EWMSC	44 (22.9)	7.39 ± 1.12	6.72 ± 0.65
SAPH	18 (9.4)	10.00 ± 1.95	5.64 ± 1.09
SGRH	9 (4.7)	8.61 ± 2.86	8.00 ± 1.45
MHWH	7 (3.7)	12.86 ± 3.38	6.93 ± 1.15

**Department at hospital**			
Paediatrics	30 (15.6)	10.00 ± 1.53	6.30 ± 0.81
Surgery	29 (15.1)	5.17 ± 0.98	5.21 ± 0.75
General Medicine	30 (15.6)	7.17 ± 1.63	5.28 ± 0.68
Obstetrics & Gynaecology	21 (10.9)	9.64 ± 1.82	5.62 ± 0.81
Psychiatry	19 (9.9)	10.53 ± 1.92	5.42 ± 1.05
Orthopaedics	10 (5.2)	6.25 ± 2.31	6.35 ± 1.23
Radiology	9 (4.7)	6.94 ± 2.49	6.78 ± 1.75

Others	24 (12.5)	5.94 ± 1.33	5.33 ± 0.75
Overall scores	192	7.77 ± 0.56	5.69 ± 0.29

Asterisks (*) show statistical significance using ANOVA. ^*1 ^p = 0.023; ^*2 ^p = 0.007.

### Assessment of acceptance

The mean acceptance score was 5.69 ± 0.29 (maximum score = 14), Table 1. Gender, nationality, country of study, hospital site, specialty and experience did not influence acceptance scores. Most physicians (60.4%) believed that herbal medicines were beneficial to health. Seventy-eight physicians (40.6%) reported that they had used this healthcare modality in the past, and 60 of these 78 respondents (or 76.9%) indicated that they were satisfied with the outcome.

Fifty-two respondents (27.1%) had previously recommended the use of medicinal herbs to their patients, and were also able to identify the herbs and herbal products in the treatment and management of diseases such as peptic ulcers, prostate enlargement and hepatitis, which supported their acceptance of this modality. However, only 14 physicians (7.3%) had ever advised their patients to consult an herbalist. Forty-three or 22.4% of the sample indicated that they would recommend medicinal herbs, if the option were available, to patients who were refractory to treatment with conventional allopathic medicines. Thirty physicians (15.6%) indicated that they accepted herbal medicines as a viable healthcare option, as they were aware of traditional medicinal practices, such as Ayuvedic medicine, and clinical trials that supported safety and efficacy of herbal remedies. Most physicians (58.3%) also expressed their willingness to allow their patients to participate in randomized controlled clinical trials to validate the safety and efficacy of medicinal herbs.

For 73 physicians (39.1%) the primary reason for rejecting herbal medicines was due to the sparse scientific information available from clinical trials to support the safety and efficacy of medicinal herbs in healthcare management. To a much lesser extent other reasons for rejecting herbal medicines included an absence of impartation of such knowledge during their medical training, non-relevance to specific specialties and the medico-legal issues of prescribing this modality in the public healthcare delivery system in Trinidad. Other physicians indicated that they personally did not believe that herbs were either safe or beneficial and one respondent commented "... and some are not scientifically proven to work and can give patients a false hope." However, most respondents (81.3%) believed that continuing education in herbal medicine was important to facilitate greater doctor-patient interaction in this mushrooming area of healthcare management.

### Assessment of knowledge

Mean knowledge score was 7.77 ± 0.56 (maximum score = 52), Table 1. Gender, hospital site or specialty did not influence knowledge; however, nationality and country of study significantly affected knowledge on medicinal herbs, p < 0.05. Tukey's test showed that physicians native to and trained in the Caribbean and Latin America had significantly higher knowledge scores than their Indian/Asian and Nigerian counterparts. A trend of moderate increase in knowledge with years of medical experience was observed, but this was not statistically significant.

Ninety-six respondents (50%) could identify at least two (2) Caribbean medicinal herbs and their traditional uses, whereas only 54 (28.1%) could identify at least two (2) non-Caribbean medicinal herbs and their uses. Most respondents were unable to identify at least one contraindication for either Caribbean (82.3%) or non-Caribbean herbs (87.5%) and only 29 physicians (15.1%) were able to correctly identify any known herb-drug interaction. Most physicians (55.7%) reported asking patients about their medicinal herb use in the history taking of drug use.

The most popular herbs identified are listed in Table 2, and include several medicinal plants of Caribbean and non-Caribbean origins such as lemongrass (*Cymbopogon citratus *DC. Stapf), noni (*Morinda citrifolia *Linn.) and saffron (*Curcuma longa *L.). Interestingly, the medicinal use of marijuana (*Cannabis sativa*) was recognized in third place with 26 physicians (13.5%) recognizing its usefulness.

**Table 2:** Most common medicinal herbs and their reputed uses cited by public health sector physicians in Trinidad

**Common name**	**Botanical name**	**Traditional medicinal uses identified**	**No. (%) of physicians citing**
Lemon grass	*Cymbopogon citratus *(DC.) Stapf.	Fever, common cold, blood purifier, headache	34 (17.7)
Gingko	*Gingko biloba *L.	Enhance memory, vertigo, dementia, impotence	29 (15.1)
Marijuana	*Cannabis sativa *L.	Asthma, glaucoma, anorexia, multiple sclerosis, epilepsy, nausea/vomiting, analgesic antidepressant	26 (13.5)
Garlic	*Allium sativum *L.	Hypertension, fever, joint pain, cough, analgesic, anti-parasitic, kidney problems	24 (12.5)
Aloes	*Aloe vera *(*Aloe barbadensis *Miller)	Superficial wounds/cuts, dysmenorrhea, acid peptic disease, acne, skin purifier, peptic ulcer, constipation	23 (12.0)
Senna	*Cassia senna *L.	Laxative, antihelmentic, 'colon cleanser', 'blood purifier'	19 (9.9)
Noni	*Morinda citrifolia *Linn.	Hypertension, acne, diabetes, pain, immune system, prostate	18 (9.4)
Ginseng	*Panax ginseng *C.A. Mayer	Enhance stamina and memory, aphrodisiac	17 (8.9)
Echinacea	*Echinacea purpurea *L. Moench	Boost immune system, upper respiratory tract infections, common cold, acne	15 (7.8)
St. John's wort	*Hypercium perforatum *L.	Depression	15 (7.8)
Evening primrose oil	*Oenothera biennis *Nutt.	Hormonal imbalance in menopause, breast pathology and mastalgia	11 (5.7)
Saw palmetto	*Serenoa repens *(Bartram) Small	Benign prostatic hypertrophy	11 (5.7)
Wonder-of-the-world	*Kalanchoe pinnata *(Lam.) Pers. l	Swelling, earache, rash, mumps, cuts	9 (4.7)
Saffron	*Curcuma longa *L.l	Purgative, antiseptic, acne common cold, fever, cough antihistamine, menstrual problems, osteoarthritis	9 (4.7)

Only 18 physicians (9.4%) had access to information on herbal medicine at their place of work, and 30 (15.6%) had ever attended conferences or workshops where a paper on herbal medicine was presented or discussed. Although 105 physicians (54.7%) were aware of herbal pharmacopoeias, only 13 of these 105 (or 12.4%) could name at least one.

## Discussion

This study showed for the first time the marked disparity between acceptance and knowledge of herbal medicines by public health sector doctors in Trinidad. The mean acceptance score was about 40% of the total possible score, whereas the mean knowledge score was about 15% of the total possible score. Generally, these healthcare providers had relatively high acceptance levels with poor knowledge. The gap between acceptance and knowledge of herbal remedies by physicians may indicate the differential between traditional/cultural beliefs and the lack of access to information.

We propose that this disparity could be partly explained by the origin and composition of Trinidad's 1.3 million inhabitants [26]. More than 80% of the population is composed of descendants of enslaved Africans and indentured Asian Indian labourers who arrived in the Caribbean during the last few centuries [27]. These two major ethnic groups now coexist in almost equal proportions on the island. There is also a minority presence of peoples of Middle Eastern, European and Chinese origin. A significant proportion of the population is comprised of individuals of 'mixed' heritage arising out of the intermarriage among all ethnic groups. These recently transplanted peoples have attempted to maintain their traditional medicinal practices, and in some cases medicinal plants were introduced into the flora of the island [28-31]. Although most Trinidadians today are aware of the benefits of some traditionally and culturally used medicinal herbs, there is generally a lack of transmission of substantial traditional knowledge from generation to generation. This progressive loss of traditional knowledge is further exacerbated by the displacement of traditional medicinal practices by Western medicine in the modern Trinidadian society. Nonetheless, most Trinidadians accept medicinal herbs as a viable option in healthcare management [15-17].

Unlike earlier studies that used closed questions, where true-or-false responses were required for a limited number of popular medicinal herbs, most of the knowledge questions in our survey instrument were open-ended. We postulated that this approach would provide a wider breadth to assess 'true' knowledge without limiting responses and also eliminate the likelihood of respondents guessing the correct answers. Besides, our finding of poor knowledge among physicians in Trinidad was consistent with other studies assessing medical practitioners [18,19] and other healthcare professionals such as nurses [32] and pharmacists [33].

Over 40% of physicians interviewed reported using medicinal herbs in the past, with more than three-fourths of these being satisfied with the outcome. Our results were markedly higher than in the Norwegian study where only 12% of physicians reported the use of alternative medicines, which included herbal medicines [5]. Our results were similar to a US study where 66% of pediatricians supported the view that complementary and alternative medicines could ameliorate symptoms or hasten recovery [34].

About one-fifth of our sample population suggested that this therapeutic option should be explored when conventional allopathic medicines fail, and this finding was similar to that reported in a recent UK study [35] where physicians' personal attributes and training influenced the likelihood of recommending herbal medicines. Hyodo et al [6] reported that 13% of oncologists noted CAM-associated improvement in their patients, and 9.9% indicated that there was sufficient evidence to support use of this modality. Most medical practitioners in Trinidad were willing to go a step further by agreeing to allow their patients to be recruited for randomized controlled clinical trials that would validate (or otherwise) the safety and efficacy of Caribbean "bush teas".

Most respondents, particularly native West Indians, were able to identify the traditional medicinal uses of Caribbean herbs; and fewer were able to identify non-Caribbean herbs and their indications. In this study, knowledge of contraindications and herb-drug interactions was very poor and was similar to a US study which also demonstrated a knowledge deficit as it related to adverse effects of herbs [36]. We noted that physicians of Indian/Asian and Nigerian origins were not familiar with medicinal herbs in the Caribbean and this adversely affected their knowledge scores. Despite this shortcoming, most physicians of Indian origin were aware of Ayuvedic and other traditional medicine practices, and of clinical trials with herbal remedies which supported their acceptance of this healthcare modality. Interestingly, many medicinal herbs presently growing in the Caribbean were transplanted by the Asian and African diaspora during the last few centuries.

More than half of the physicians interviewed reported having asked their patients specifically about herbal medicine use when taking a drug history, and this was similar to an Israeli study where 58% of physicians always or frequently asked their patients about their use of complementary medicine [37]. Our results were significantly higher than the 20% of pediatricians in a US study who queried use in their patients [34]. We did not determine whether this information was recorded in the patients' charts or whether physicians attempted to advise or dissuade patients from using this modality. Cohen et al [38] showed that although physicians may have asked their patients about herb use, only about one-third of these doctors actually documented this information in patients' charts. The subsequent impact of this query on the quality of healthcare is unknown.

As the use of medicinal herbs continues to increase worldwide, there has been a parallel surge in research to isolate pharmacologically active pure compounds from medicinal plants, and clinical investigations are being done to establish the safety profile and efficacy of some of the more popular traditionally used herbal remedies [39-42]. The reports of these investigations are appearing in several international herbal pharmacopoeias including the European Scientific Cooperative On Phytotherapy (ESCOP) [20], the German Commission E [21] and TRAMIL [22] giving such information as botanical names, common names, traditional uses and therapeutic indications, chemical constituents, contra-indications and pharmacological properties of selected herbs. Evidence-based information is available and could be compiled and structured in a format to impart workable knowledge to medical students and practicing physicians. The availability of these herbal medicine resources at the worksite, as reference material, is crucial for creating an environment conducive to more efficient physician-patient interaction in the area.

At present, formal training in herbal medicine at the regional university (The University of the West Indies) where most of our physicians are trained, does not form part of the curriculum and subsequently graduates possess no formal knowledge in the area. Our study showed that most respondents agreed that continuing education in herbal medicines was necessary for effective patient consultations. Recent studies have shown that herbal medicine educational interventions taught as structured programs via different media significantly improved physicians' knowledge, confidence and their interactions with patients [18,43]. Frenkel et al [44] showed that 72% of family practice physicians who participated in a structured patient-centred educational program on CAMs reported significant positive attitudinal changes.

Our survey was conducted at public hospitals throughout Trinidad, and obviously excluded physicians who worked exclusively at private institutions and probably different responses would have been elicited from these physicians. Although the use of quota sampling with stratification was advantageous for our research purposes, interviewer bias may have been introduced by the non-random convenient selection of respondents, resulting in a sample that may not have been truly representative. Although open-ended questions were especially useful for gathering responses on knowledge, where replies were too numerous to code (for example, more than 45 medicinal plants and several traditional indications were identified in this study), there were some limitations. Open-ended questions require more thought and are taxing on the respondent and this may have affected the quality of responses. Also, responses to these open-ended items may have been summarized and the true meaning distorted by the interviewer or by the coding process used for data entry.

Notwithstanding these limitations, the burden of our results suggests that there is an urgent need for educational intervention with regard to herbal medicine in the training of our physicians. We propose that there be an integration of herbal medicine into the current medical curriculum so that future physicians would be better able to communicate with their patients on this healthcare modality. Continuing education programs are also recommended so that practicing physicians would have the opportunity to upgrade their knowledge in this rapidly expanding area of significant public health concern. In the interim, public health institutions should be equipped with reputable herbal pharmacopoeias and electronic databases to answer questions that would arise during the course of clinical practice.

## Conclusion

Our findings showed that medical practitioners in the public healthcare sector in Trinidad generally accepted herbal remedies as a viable option although they lack sufficient knowledge on the uses and potential risks associated with this modality. This result directly contradicted our initial hypothesis that herbal remedies would be rejected (or poor acceptance) and that this would correlate with poor knowledge. This creates an interesting scenario where the gap between acceptance and knowledge provides an ideal opportunity to facilitate the introduction of educational programs and policies that would increase the knowledgebase of these healthcare professionals. Well-informed physicians would be more confident in their interactions with patients and this would improve the quality of healthcare delivery, as more meaningful communication on important issues such as adverse effects and herb-drug interactions would be facilitated. The increasing trend in the use of herbs is set to continue well into the foreseeable future and the enhanced knowledgebase of physicians would redound to the benefit of patients who would appreciate a non-judgmental environment when discussing healthcare needs.

## Appendix 1. The eight *weighted *acceptance questions (Total score = 14 points)

1. Do you believe that herbal medicines are beneficial in healthcare management? (2 points for affirmative answer)

Yes □

No □

2. Have you ever recommended the use of herbal medicines? (2 points for affirmative answer)

Yes □

No □

3. Have you ever prescribed herbs to patients for medicinal purposes? (2 points for affirmative answer)

Yes □

No □

4. Have you ever recommended patients to an herbalist? (2 points for affirmative answer)

Yes □

No □

5. Have you ever personally used herbs? (2 points for affirmative answer)

Yes □

No □

6. Do you think that the use of herbal medicines should be limited only to patients who have failed conventional therapy? (1 point for affirmative answer)

Yes □

No □

7. Do you think that continuing education in herbal medicines is important? (1 point for affirmative answer)

Yes □

No □

8. Would you be willing to allow your patients to participate in clinical trials to evaluate the efficacy of Caribbean 'bush' teas? (The normal trial protocol has been followed) (2 points for affirmative answer)

Yes □

No □

## Appendix 2: The ten *weighted *knowledge questions (Total score = 52 points)

1. Can you identify any five (5) Caribbean herbs and their common usages? (2 points for each herb and its correct use; 10 points total)

2. Can you list five (5) contraindications of named Caribbean herbs? (2 points for each herb and its correct contraindication(s); 10 points total)

3. Can you identify any five (5) imported herbs and their common usages? 2 points for each herb and its correct use; 10 points total)

4. Can you list five (5) contraindications of named imported herbs? (2 points for each herb and its correct contraindication(s); 10 points total)

5. Can you list any two (2) herb-drug interactions? (2 points for each correct answer; 4 points total)

6. Do you specifically ask your patients about their use of herbal medicine when taking a drug history? 1 point for affirmative answer)

Yes □

No □

7. Are you aware that several international herbal pharmacopoeias exist? (1 point for affirmative answer)

Yes □

No □

8. If yes, could you give at least one (1) example of a herbal pharmacopoeia? (3 points for correct pharmacopoeia)

9. Are you aware that there are several completed and ongoing international clinical trials on the efficacy and safety of herbal medicines? (1 point for affirmative answer)

Yes □

No □

10. If yes, could you identify one example? (2 points for correct example)

## Competing interests

The author(s) declare that they have no competing interests.

## Authors' contributions

YNC was the P.I. and was responsible for the study concept, development of methodology, coordinating of the research activities, analyzing the data, and writing the manuscript. KK, TB, SB, MF and KN were involved in methodological development, data collection, data input and analysis. OM was involved in methodological development. AFW was involved in data input and statistical analysis. CES was involved in an advisory capacity in the study concept and development of methodology phases of the research. All authors read and approved the final manuscript.

## Pre-publication history

The pre-publication history for this paper can be accessed here:


